# HIV incidence in men who have sex with men in England and Wales 2001–10: a nationwide population study

**DOI:** 10.1016/S1473-3099(12)70341-9

**Published:** 2013-04

**Authors:** Paul J Birrell, O Noel Gill, Valerie C Delpech, Alison E Brown, Sarika Desai, Tim R Chadborn, Brian D Rice, Daniela De Angelis

**Affiliations:** a MRC Biostatistics Unit, Institute of Public Health, Cambridge, UK; b Health Protection Agency, Health Protection Services, London, UK

## Abstract

**Background:**

Control of HIV transmission could be achievable through an expansion of HIV testing of at-risk populations together with ready access and adherence to antiretroviral therapy. To examine whether increases in testing rates and antiretroviral therapy coverage correspond to the control of HIV transmission, we estimated HIV incidence in men who have sex with men (MSM) in England and Wales since 2001.

**Methods:**

A CD4-staged back-calculation model of HIV incidence was used to disentangle the competing contributions of time-varying rates of diagnosis and HIV incidence to observed HIV diagnoses. Estimated trends in time to diagnosis, incidence, and undiagnosed infection in MSM were interpreted against a backdrop of increased HIV testing rates and antiretroviral-therapy coverage over the period 2001–10.

**Findings:**

The observed 3·7 fold expansion in HIV testing in MSM was mirrored by a decline in the estimated mean time-to-diagnosis interval from 4·0 years (95% credible interval [CrI] 3·8–4·2) in 2001 to 3·2 years (2·6–3·8) by the end of 2010. However, neither HIV incidence (2300–2500 annual infections) nor the number of undiagnosed HIV infections (7370, 95% CrI 6990–7800, in 2001, and 7690, 5460–10 580, in 2010) changed throughout the decade, despite an increase in antiretroviral uptake from 69% in 2001 to 80% in 2010.

**Interpretation:**

CD4 cell counts at HIV diagnosis are fundamental to the production of robust estimates of incidence based on HIV diagnosis data. Improved frequency and targeting of HIV testing, as well as the introduction of ART at higher CD4 counts than is currently recommended, could begin a decline in HIV transmission among MSM in England and Wales.

**Funding:**

UK Medical Research Council, UK Health Protection Agency.

## Introduction

High levels of HIV testing combined with prompt antiretroviral therapy (ART) might substantially reduce HIV transmission, especially if high levels of engagement and retention in care can bring about a large reduction in community viral load (the aggregate viral load within a given population or risk group).^1^, ^2^, ^3^, ^4^ Various models have investigated the effect of different thresholds for the initiation of treatment, treatment uptake, and adherence rates and suggest that widespread ART coverage could have a population-level prevention effect, thereby reducing HIV transmission.^5^, ^6^, ^7^, ^8^

Two studies of routinely collected surveillance data have shown an association between increases in ART coverage and declines in new HIV diagnoses and community viral load, one in British Columbia, Canada,^3^ and another among men who have sex with men (MSM) in San Francisco, USA.^1^ The authors concluded that these results support the postulated secondary population-level benefits of ART. In England and Wales, MSM have the highest prevalence of HIV (9% in London and 3% elsewhere).^9^ Most MSM are tested and diagnosed in free and confidential dedicated sexually transmitted infection (STI) clinics. Link to HIV care after diagnosis is prompt (more than 95% within 3 months)^10^ and retention in care is high (more than 95% a year).^11^ The initiation of ART is recommended in patients with a CD4 count below 350 cells per μL.^12^ New diagnoses in MSM have continued to rise over the past decade despite the high and rising coverage of ART.

Although trends in new diagnoses remain a crucial measure of the HIV epidemic, they are a suboptimum measure of changes in HIV incidence.^13^ An HIV diagnosis is the outcome of three interacting processes: transmission, infection progression, and diagnosis. Consequently, the number of new diagnoses is a dynamic mixture of long-standing and recent infections, and is, therefore, not necessarily synonymous with the number of new infections. Only by reconstructing the complex mechanism underlying observed data can we disentangle the contribution of changes in testing patterns and in incidence to the recorded trends in HIV diagnoses and interpret them appropriately. We used a novel and simple CD4-staged back-calculation approach, incorporating CD4 counts at diagnosis and information on the natural history of HIV infection, to simultaneously estimate HIV incidence and trends in diagnosis rates in MSM in England and Wales for the decade 2001–10. From these estimated trends, we derived estimates of the number of undiagnosed infections over time and trends in the time from infection to diagnosis, giving additional insight into the effect of HIV testing practices over the past decade. We present model outputs alongside comprehensive data for HIV testing rates and ART coverage to examine whether increases in testing rates and ART coverage in England and Wales have corresponded to the control of HIV transmission in MSM, where control is defined as a sustained decline in incident HIV infections.

## Methods

### Data for the back-calculation model

The model uses reports of new HIV and AIDS diagnoses and information on CD4 counts around diagnosis among MSM in England and Wales. Specifically, the model uses quarterly aggregated counts of new AIDS-free and late HIV diagnoses from the early 1980s to the end of 2010. AIDS-free HIV diagnoses are those with no accompanying clinical AIDS diagnosis within 3 months, whereas a late HIV diagnoses refers to a clinical AIDS diagnosis that occurs before or within 3 months of HIV diagnosis. From 1991, the national HIV database has been linked (using soundex code,^14^ date of birth, and sex)^15^ to CD4 count laboratory reports from haematology laboratories.^16^ From this linkage, data are available for the distribution of CD4 counts at diagnosis. Here, a CD4 count is interpreted to be at diagnosis if it is the first recorded CD4 count for a patient taken within 3 months of their initial diagnosis. Between 1991 and 2010 these counts are available in 76% of all new diagnoses, rising to 91% in 2010.

### Other data sources

Coverage of ART, defined as the proportion of all people in HIV care who received ART at the date they were last seen, was obtained from the annual census of patients accessing care in all HIV outpatient services in England and Wales. Information relating to ART is complete for 99% of patients.^11^, ^17^ HIV testing data for MSM attending all STI clinics in England and Wales were extracted from quarterly clinic returns for the years 2001–10.^18^

The high completeness of surveillance data is ensured through mutual supplementation of information between different systems; additionally, data are necessarily of high quality, because surveillance data directly inform the commissioning and funding of HIV services. Annual loss to follow-up rates for MSM accessing HIV care are very low (<5%).^11^

### The CD4 staged back-calculation HIV transmission model

The processes of infection, disease progression, and diagnosis of HIV-infected MSM are described through a discrete multistate model, with time steps every calendar quarter and disease states defined by CD4 count and diagnosis status (appendix).^19^ After infection, transition between CD4 states is expressed through assumed known parameters representing the proportion of individuals progressing to the next CD4 state in each quarter, informed from the analysis of longitudinal CD4 counts in HIV-infected people for whom the date of infection is well estimated.^20^ In each quarter, a CD4-specific proportion of MSM is diagnosed. These proportions are allowed to vary over calendar time to reflect changing testing patterns throughout the epidemic. Using the data described above, the model permits the simultaneous estimation of HIV incidence and quarterly proportions diagnosed, from which we can quantify the changes in the time-to-diagnosis (appendix) and estimate the number of undiagnosed infections in each CD4 state, both with appropriate statements of uncertainty, expressed in terms of 95% credible intervals (CrI).

### Role of the funding source

The sponsor of the study had no role in study design, data collection, data analysis, data interpretation, or writing of the report. The corresponding author had full access to all the data in the study and had final responsibility for the decision to submit for publication.

## Results

**Figure 1:**
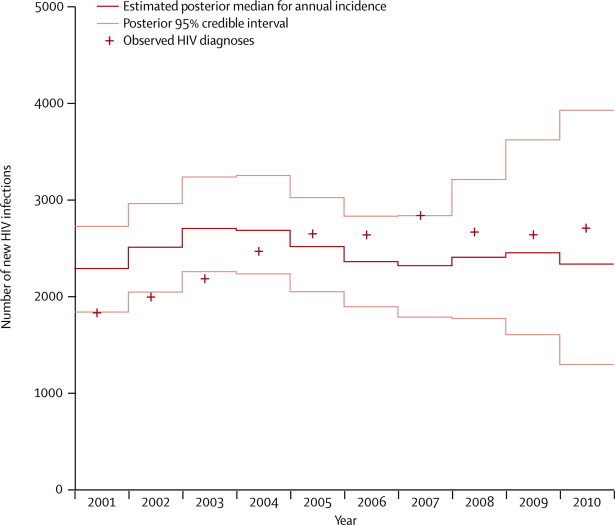
Annual numbers of new HIV infections for men who have sex with men in England and Wales

Between 2001 and 2010, the estimated number of new infections oscillated between 2200 and 2800 infections a year (figure 1). Incidence increased to a peak in 2003–04, but, after a slight decline, stabilised at 2300–2500 infections a year from 2006 to the end of 2010. From 2007 onwards, the CrIs attached to the estimates of incidence are of steadily increasing width. This uncertainty arises because the data are less informative about the most recent infection numbers due to only a small proportion of these infections having had sufficient time to be diagnosed and thus appear in the data. However, there is no statistically significant change in incidence between any 2 years over the interval and no suggestion of an increasing or decreasing trend (see appendix).

**Figure 2:**
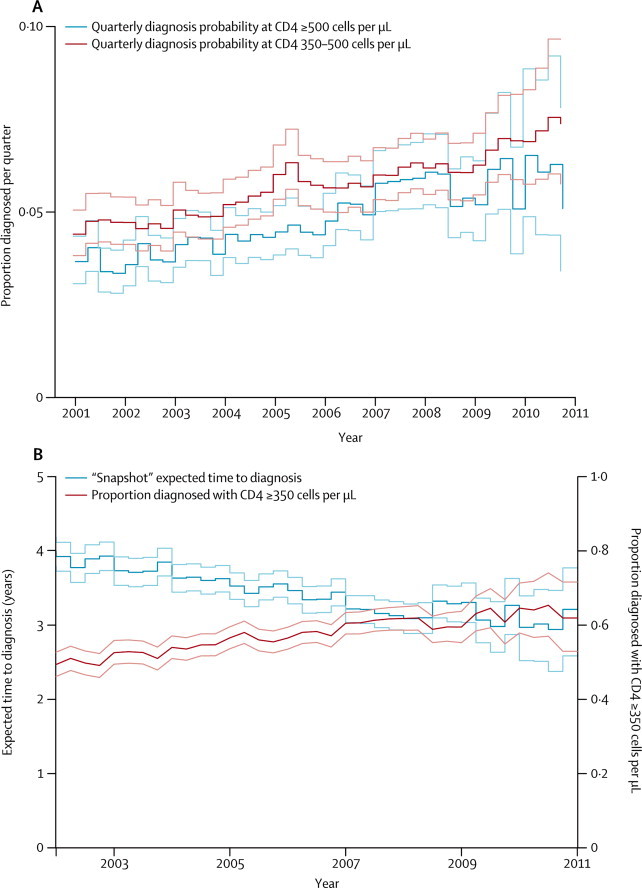
Estimated trends in diagnosis by CD4 count Proportions of HIV-infected MSM in each of the two high CD4 count states that are diagnosed per quarter (A). Estimated expected time to diagnosis and proportion diagnosed at CD4 count of more than 350 (B). Lighter coloured lines indicate 95% credible interval.

The proportion of undiagnosed individuals who are diagnosed each quarter from the two high CD4 count states (≥350 cells per μL) increased steadily over time (figure 2), with a similar trend seen for the lower CD4 counts (results not shown). As a result, the estimated snapshot time-to-diagnosis distributions (see appendix) indicate a trend towards earlier diagnosis over the decade (figure 2). The mean time-to-diagnosis interval declined from 4·0 years (95% CrI 3·8–4·2) at the beginning of 2001 to 3·2 years (2·6–3·8) by the end of 2010 (figure 2). Furthermore, the total proportion of MSM diagnosed with a CD4 count greater than the recommended treatment threshold (≥350 cells per μL) increased from a low of 48% (45–52%) in the fourth quarter of 2001 to a high of 65% (57–74%) in the third quarter of 2010 (figure 2B). Despite these developments, at 2010 rates of diagnosis, 38% of new infections will not be diagnosed until after the time at which they would have qualified for treatment (figure 2).

**Figure 3:**
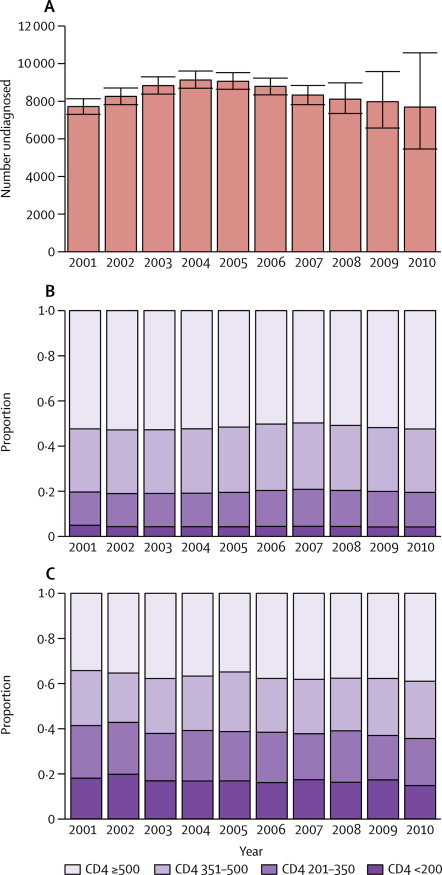
Undiagnosed infections and CD4 counts at diagnosis Median numbers of undiagnosed infections among MSM in England and Wales (A). CD4 count among undiagnosed infections in MSM (B) and newly diagnosed infections (C).

The estimated number of undiagnosed HIV infections increased steadily from the beginning of 2001 to a peak of 9140 (95% CrI 8720–9620) infections in the first quarter of 2005 (figure 3). Since this time, there has been a quarter-on-quarter decline in the estimated number of undiagnosed infections, although, similarly to the estimation of incidence, these estimates, from 2007 onwards, are characterised by increasing uncertainty. By the end of 2010 the estimated number of undiagnosed infections in MSM was 7690 (95% CrI 5460–10 580), similar to the 7370 (6990–7800) 10 years earlier.

The estimated CD4 count distribution across the undiagnosed HIV infections has changed very gradually over time (figure 3). Undiagnosed HIV infections with a CD4 count of 500 cells per μL or higher consistently account for around 50% of the undiagnosed population, with the general trend following the number of incident infections in each year. These distributions contrast starkly with the observed CD4 count distributions at diagnosis, which are more evenly distributed across the CD4 count states, although diagnoses with CD4 counts higher than 500 cells per μL are still the most common (figure 3). At the other end of the scale, the estimated number of undiagnosed HIV-infected MSM with a CD4 count of less than 350 cells per μL (and thus, in principle, eligible for treatment) remains unchanged from 1490 (95% CrI 1410–1570) at the start of 2001, to 1490 (1240–1770) at the end of 2010, although this change does represent a decrease from a peak in 2007 of 1790 (1700–1880) undiagnosed infections.

These trends in HIV incidence and prevalence can be interpreted against contemporaneous data for uptake of ART, HIV tests done in STI clinics, and numbers of new HIV diagnoses among MSM (table). The annual number of new diagnoses rose from 1640 in 2001 to 2450 in 2005 and levelled off at around 2500 in 2010, whereas the number of men who presented with clinical AIDS at the time of their HIV diagnosis remained fairly stable (125 in 2001 to 177 in 2010). The proportion of diagnoses occurring at high CD4 counts (≥500) has increased over the past 10 years, though not substantially (figure 3). Around 16 000 men were tested for HIV in STI clinics in 2001, a figure that increased 3·7 fold over the decade to 59 300, a substantial growth in testing effort (table). The number of HIV-diagnosed MSM receiving care rose steadily from 12 500 in 2001 to 27 900 in 2010, and the proportion receiving ART, already at 69% in 2001, increased to 80% in 2010. In 2001, 75% of MSM with a CD4 count of less than 350 cells per μL were in receipt of ART, compared with 84% in 2010.

**Table:** Recent trends in the number of HIV-infected men who have sex with men in England and Wales

	**2001**	**2002**	**2003**	**2004**	**2005**	**2006**	**2007**	**2008**	**2009**	**2010**
Total number of infected individuals in HIV care	12 529	13 834	15 354	17 028	18 877	20 762	22 683	24 342	26 061	27855
Total number of infected individuals receiving ART	8643	10 029	10 622	11 695	12 866	14 731	15 907	18 150	19 999	22399
Percentage of infected individuals in HIV care in receipt of ART	69%	72%	69%	69%	68%	71%	70%	75%	77%	80%
Number of HIV tests in STI clinics	16 000	18 200	24 000	26 400	30 700	35 000	37 200	42 700	52 300	59 300
New HIV diagnoses	1640	1780	1970	2240	2450	2440	2640	2470	2440	2550
New HIV diagnoses presenting as AIDS	158	175	164	177	170	153	170	157	129	152

ART=antiretroviral therapy. STI=sexually transmitted infection.

## Discussion

Our analysis of 20 years of a comprehensive population-wide surveillance dataset using a CD4-based back-calculation model shows that, over the past decade, there is no evidence of a decline in incidence among MSM, with new infections continuing at around 2300–2500 a year. There is a significant declining trend in the estimated number of undiagnosed HIV infections between a peak at the end of 2004 to the end of 2008, and there is the suggestion that this trend may continue into 2010 (figure 3A), although the uncertainty attached to the most recent estimates makes this decrease difficult to detect. By the end of 2010 we estimate that around 7700 MSM with HIV remained undiagnosed, corresponding to 22% of the total number of MSM with HIV, a decrease from 37% of the total number of infections in 2001.

Our results confirm previous speculations about the high level of continuing HIV transmission in MSM and are consistent with earlier estimates of undiagnosed prevalence obtained using a multiparameter evidence synthesis approach, where a complex dynamic model is used to synthesise the information contained in several datasets.^21^, ^22^ Over the past decade, the estimated diagnosed proportions from each CD4 state all steadily increased, resulting in a shortening of the mean time to diagnosis by 20% from 4·0 to 3·2 years. Through our back-calculation approach we estimated that, since 2001, the CD4 count of almost a third of the undiagnosed individuals was between 350 cells per μL and 500 cells per μL, and in half the count was above 500 cells per μL.

Our modelling approach has several advantages. First, the model takes into account the interaction of three distinct processes (infection transmission, progression, and diagnosis), allowing the estimation of both rates of infection and diagnosis. Ignoring the propensity for individuals to be diagnosed, and temporal changes in this propensity, will lead to estimates for incidence that are based solely on CD4 cell counts and the natural history of HIV infection. Such estimates will typically be biased because of an overestimation of the time since infection as the time at risk of a diagnosis is completely ignored. Second, estimates of the number of undiagnosed infections in the various CD4 states are automatically generated and are consistent with the available information, without need for simplifying, untestable, assumptions (eg, similarity of the CD4 population profile of newly diagnosed and undiagnosed infections). Third, we use observed data from multiple sources, thus reducing the likelihood of biases that might be inherent in individual databases. Other researchers have also made use of multiple data sources, but they have referred to situations where, unlike here, there was no linkage between the new HIV and AIDS diagnoses,^23^ the data sources have been used independently with a comparison of the resulting estimates,^24^ or the additional data are simply used for model validation.^25^ Finally, the coherent, joint estimation of the time-varying incidence and diagnosis rates obtained through applying our back-calculation method strengthens the argument that CD4 counts at diagnosis should be an integral part of national HIV surveillance systems. The availability and completeness of these data in new diagnoses databases in developed countries are poor.^26^

This work represents only the first step of a more comprehensive model, which could incorporate many more complexities (eg, a primary infection stage, or heterogeneity in risk behaviours), allowing the comprehensive investigation of scenarios that might explain the lack of transmission control. Some relevant ideas have already been explored in the analysis of the Dutch HIV epidemic.^25^

In conclusion, although there is incontestable evidence regarding the benefits of treatment as prevention at an individual level, there is no indication so far that the increased rates of testing and widened access to treatment have controlled HIV transmission in MSM in England and Wales (panel). The most plausible explanation for lack of HIV transmission control (ie, the lack of any sustained decline in incidence) during this period is a resurgence in unsafe sexual behaviour (largely because of treatment optimism),^27^ and insufficiently frequent HIV testing among this population. The resurgence in unsafe sexual practices is evident through a concomitant epidemic of bacterial STIs and may have been amplified through social media accelerating wider partnership formation.^18^, ^28^, ^29^ There is strong evidence of a multiple-fold increase over time in the numbers of HIV tests administered in STI clinics, the preferred facility for testing for 77% of high-risk MSM,^30^ and where 93% of MSM receive their initial HIV diagnosis (S Croxford, Health Protection Agency, personal communication). However, this increased testing effort might not have sufficiently improved coverage of testing across the MSM population and the relatively modest 20% decrease in the estimated expected time-to-diagnosis and the 15% of diagnoses each year that seem to be clinically driven (diagnosis with a CD4 count of less than 200 cells per μL, figure 3) would seem to confirm this. This improvement in diagnosis is certainly insufficient to be able to capture a greater proportion of primary infections, which have been estimated to be responsible for up to 50% of transmission.^31^, ^32^

Panel **Research in context**

**Systematic review**

A search of PubMed using (“antiretroviral therapy” OR “HIV testing”) AND (“HIV diagnoses” OR “HIV incidence” OR “HIV prevalence”) AND “HIV prevention” yielded 19 articles. We selected a subset of these articles, choosing those that investigated the (in some cases, potential) impact of expanded testing and/or treatment upon HIV incidence and that included a significant mathematical or statistical modelling component.^1^, ^3^, ^5^, ^7^ These modelling studies were augmented by browsing related citations, identifying three further modelling studies.^6^, ^8^, ^25^ Since this is a highly active area of current research, further references came to our attention during the lifespan of this work.^2^, ^4^, ^13^ None of these studies were directly relevant to a health-care system like that of England and Wales, where HIV is hyperendemic, retention in care is high and treatment is prescribed to many HIV infected individuals with a CD4 count less than 350 cells per μL.

**Interpretation**

Using the rich array of HIV surveillance data available in England and Wales, we have adopted a novel, relatively parsimonious, CD4-based back-calculation model to estimate simultaneously HIV incidence and CD4-specific diagnosis rates among MSM over the period 2001–2010.^19^ Furthermore, this work results in the estimation of the number of undiagnosed infections by CD4 count. We did not find any clear evidence for declining incidence despite an estimated shortening of the time-to-diagnosis from 4·0 years to 3·2 years. The number of undiagnosed infections declined only slightly from the middle of the decade, returning to 2001 levels. These results are set alongside data highlighting expanded testing effort and greater treatment coverage over the period. Modelling studies have raised great enthusiasm for the potential of test-and-treat practices to provide control of HIV transmission.^1^, ^2^, ^3^, ^4^, ^5^, ^6^, ^7^ However, a few studies have identified situations where such practices have only a modest or short-lived benefit and discuss their limitations.^8^, ^13^, ^25^ In this work we demonstrate how expanding testing and high (and rising) treatment coverage have not corresponded to a decline in HIV transmission. We suggest that healthcare services in England and Wales will need to provide more targeted testing of the at-risk population and to start treatment earlier if a sustained decline in HIV incidence is to be brought about.

Treatment status and viral load are available for every patient in care in England and Wales, in which almost all diagnosed individuals are retained. By 2010, 80% of all diagnosed infections were being treated with antiretroviral therapy, rising to 84% in those with a CD4 count lower than 350 cells per μL. Recent evidence shows that around 35% of patients, most of whom are not in receipt of treatment, have viral loads of greater than 1500 copies per mL and are therefore at risk of transmitting the infection.^33^ By contrast with the treatment cascade in the USA, where there is a lower retention in care, albeit with a higher proportion of these patients on antiretroviral therapy,^34^ our estimates of the number of undiagnosed MSM in England and Wales are higher than the observed number of diagnosed MSM who remain untreated (table). This finding strongly suggests that the undiagnosed infections represent the principal part of the community viral load reservoir driving HIV transmission.^35^, ^36^

The findings in our paper should alert policy makers and public health authorities to the limited effect of the national HIV Strategy (England),^37^ which aimed to reduce transmission through increasing the uptake of HIV testing in STI clinics, and to the fact that high antiretroviral coverage alone might not be sufficient to eliminate HIV transmission. Primary prevention and earlier, more targeted, testing must continue to be prioritised as part of any national HIV prevention plan. In view of the profile of CD4 counts in newly diagnosed patients, showing that around 60% are not immediately eligible for antiretroviral therapy, the initiation of treatment on diagnosis of HIV infection, irrespective of CD4 count, might well be necessary to achieve control of HIV transmission. To this extent, we welcome the new British HIV Association guidelines recommending that clinicians discuss the benefits of early treatment uptake as a prophylactic to protect sexual partners.^12^
