# Supplementary material for: HIV incidence in men who have sex with men in England and Wales 2001–10: a nationwide population study
Source: Lancet Infect Dis. 2013 Apr;13(4):313–8. doi: 10.1016/S1473-3099(12)70341-9 (PMC3610092; doi:10.1016/S1473-3099(12)70341-9)
Supplement: Supplementary appendix [file mmc1.pdf]

THE LANCET Infectious Diseases

Supplementary webappendix

This webappendix formed part of the original submission and has been peer reviewed. We post it as supplied by the authors.

Supplement to: Birrell PJ, Gill ON, Delpech VC, et al. HIV incidence in men who have sex with men in England and Wales 2001–10: a nationwide population study. *Lancet Infect Dis* 2013; published online Feb 1. http://dx.doi.org/10.1016/S1473-3099(12)70341-9.

Trends in HIV incidence among men who have sex with men in England and Wales in the era of increased HIV testing and treatment: a nationwide population study, 2001 to 2010

Technical Appendix

Results presented in the main text were obtained from the implementation of a Bayesian back-calculation model based on counts of new diagnoses of HIV and/or AIDS, stratified by CD4 count.

Back-Calculation

Back-calculation permits infection incidence to be estimated from time series of observed counts of disease endpoints and information on the distribution of the time from infection to the endpoint of interest. If time is divided into intervals $(t_{i-1}, t_i]$, $i = 1, ..., I$, in the discrete case, the method is based on the equation:

$$\mu_i = \sum_{j=1}^{i} h_j f_{i-j}$$

where μ_i is the expected number of endpoint events in the ith time interval, h_j is the expected number of new infections in the jth time interval and f_{i-j} represents the probability that the time between the infection and the endpoint is equal to $i - j$ time intervals.

Back-calculation was first used to estimate the incidence of HIV using AIDS diagnoses as an endpoint,[1] and the method has been subsequently extended, replacing AIDS diagnoses with HIV diagnoses.[2;3] However, the occurrence of an HIV diagnosis, unlike AIDS, is not simply a function of infection progression. Diagnosis of HIV-infection follows the processes of infection transmission, infection progression, and presentation for, and acceptance of, testing. These processes interact in a complex way. Only by relating the observed data through a suitable back-calculation framework is it possible to unravel the effects of changes over calendar time in HIV testing patterns and in underlying HIV incidence. In the absence of direct knowledge of this shifting time to HIV diagnosis distribution, additional information is required to estimate both this distribution and infection incidence. Such information could be an indicator of recent infection or levels of some prognostic

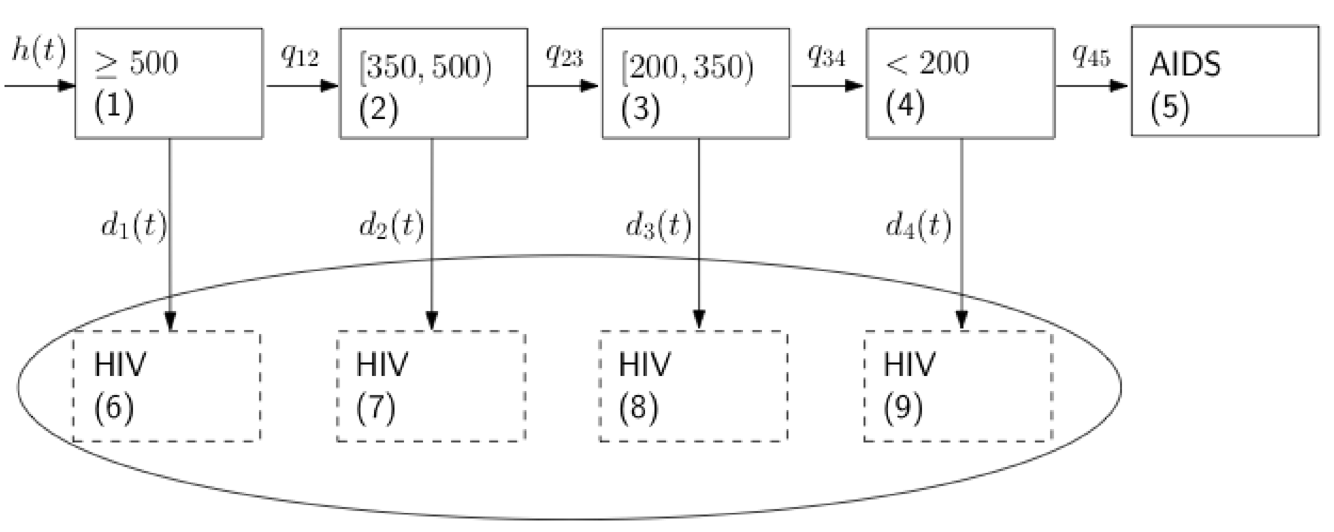

Figure 1*: A multi-state model for population-level CD4 progression, leading to HIV and AIDS diagnoses.*

marker at diagnosis.[4;5] An earlier multi-state model was adapted and developed to represent the processes that underlie the observed HIV diagnosis data,[5] which incorporated data on CD4 count at HIV diagnosis, and allowed the time-to-diagnosis to vary as infection progressed between disease stages defined by CD4 count (Figure 1).

New infections occur according to a (non-homogeneous) Poisson process with h_i representing the expected number of new infections arriving in stage 1 during the interval $(t_{i-1}, t_i]$. At time t_i, the expected number of individuals in each CD4 stage, k, $k = 1, ..., 4$, is given by E_{ki}. Of these, a proportion $d_{k(i+1)}$ will be diagnosed in the next time interval $(t_i , t_{i+1}]$. Of those remaining undiagnosed, a proportion $q_{k(k+1)}$ will progress to the next CD4 stage in the subsequent time period. The time-to-diagnosis distribution is, therefore, a complex function of the diagnosis probabilities and the disease progression parameters. The disease progression parameters are assumed fixed and known from an analysis of CASCADE data.[6]

As a result of the assumptions on the infection process, the number of arrivals into stages 6-9 (HIV diagnoses) and 5 (AIDS diagnoses) during the ith interval, are Poisson distributed with means μ_i^{HIV} and μ_i^{AIDS} respectively. These means are evaluated through a recursive process:

$$\mu_i^{HIV} = \sum_{k=1}^{4} E_{k(i-1)} d_{ki}$$
$$\mu_i^{AIDS} = E_{4(i-1)} \left(1 - d_{4i}\right) q_{45}. \tag{1}$$

Further technical detail can be found in a complementary paper [7].

The expected proportions of the HIV diagnoses in the ith interval that are attributable to individuals from CD4 state $k = 1, . . . , 4$, say r_{ki}, can then easily be found by

$$r_{ki} = \frac{E_{k(i-1)} d_{ki}}{\mu_i^{\mathrm{HIV}}} \tag{2}$$

Data

Data are available on both the total number of HIV diagnoses and AIDS diagnoses for the entire history of the HIV epidemic in England & Wales. The first diagnosis occurred in mid-1979, so t_0 was set to be the beginning of 1978, to allow sufficient incubation time for this initial diagnosis. Each time-step corresponds to a quarter year (three months). If an AIDS diagnosis occurred within one quarter of an initial HIV diagnosis, it was assumed that the diagnosis was of AIDS and not of HIV (and the individual contributed to the count at state 5 and not at state 9). Similarly, CD4 counts were assumed to be "at diagnosis" if they were measured within one quarter of the initial HIV diagnosis. This CD4 count data was available from 1991 onwards, with 25% of diagnoses having CD4 data in 1991 rising to 91% in 2010.

Although both types of diagnosis (AIDS and HIV) were available throughout the entire history of the HIV epidemic, a diagnostic test for HIV became widely available only in 1984 and very few diagnoses were prior to this year, mostly due to the retrospective testing of archived specimens.

Bayesian Inference

A Bayesian framework for statistical inference was adopted for the ease with which it incorporates multiple datasets and can augment data with additional information on model parameters through prior probability distributions. Bayesian inference combines the information held in the priors with that held in the data, via the likelihood function, to produce posterior distributions for model parameters. These distributions form the basis of our inferences.

Likelihood

The overall likelihood arises as the product of the component likelihoods for the observed quarterly HIV diagnosis counts,

X_i^{HIV}, the quarterly AIDS diagnosis counts, X_i^{AIDS}, and the quarterly CD4 count at diagnosis data $\boldsymbol{W}_i$.

$$L\left(X^{\mathbf{HIV}}, X^{\boldsymbol{AIDS}}, W\right) = \left(\prod_{i=1}^{I} L\left(X_i^{\text{HIV}}\right)\right)\left(\prod_{i=1}^{I} L\left(X_i^{\text{AIDS}}\right)\right)\left(\prod_{i=1}^{I} L\left(W_i\right)\right).$$

The likelihood for the diagnosis counts arise from the fact that these are independent Poisson random variables, i.e.

$$\begin{aligned} X_i^{\text{HIV}} &\sim \text{Po}\left(\mu_i^{\text{HIV}}\right) \\ X_i^{\text{AIDS}} &\sim \text{Po}\left(\mu_i^{\text{AIDS}}\right). \end{aligned} \tag{3}$$

The CD4 count data are based on a sub-sample of the diagnoses of known size, $N_i \leq X_i$, giving the number of these diagnoses that fall into each of the CD4 count strata. If W_{ki} is the number of diagnoses in the ith interval with a CD4 count in CD4 state k, then $\boldsymbol{W}_i = (W_{1i}, ..., W_{4i})$ follow a multinomial distribution

$$W_i \sim \text{Mn}\left(N_i, r_i\right),$$

where r_i is the vector of probabilities $(r_{1i}, ..., r_{4i})$ as defined in (2).

Priors

If both the infection incidence process, $\{h_i : i = 1, ..., I\}$ and diagnosis process $\{d_{ki} : i = 1, ..., I; k = 1, ..., 4\}$ are allowed to freely vary at each time, then the model is over-parameterised and estimation is difficult. Therefore, in the interests of model parsimony and identifiability, some smoothing of these processes is employed. This is done by assuming that they are both vary according to a random walk on the log- and the logistic- scales respectively. The random walks ensure that sudden jumps do not occur and that, for example, incidence in one quarter is correlated to incidence in the next quarter. For example, in the case of incidence, if $\gamma_i = \log(h_i)$, then

$$\gamma_{i+1} \sim N\left(\gamma_i, \sigma_{\gamma,i}^2\right).$$

These random walks introduce variance parameters that control the range of likely values for the step size, e.g. $\sigma_{\gamma,i}^2$ above. For the random walk on the diagnosis probabilities, this variance is held fixed over time whereas for the expected incidence, there is a breakpoint early on in the epidemic (in 1986) to represent the end of a period of initial early growth. These random-walk specifications for both the time-varying rates of infection and diagnosis describe the prior probability distributions for the majority of model parameters. All that is required beyond this is the specification of prior probability distributions for the variance parameters and the starting point for each random walk. All variance parameters are chosen to have reasonably uninformative priors, whereas the prior for the initial level of incidence is focused on very small values.

Because of the timing of the introduction of the diagnostic test for HIV infection, the random-walk process governing the diagnosis probabilities is only considered to have begun from 1985 onwards, once the use of an HIV diagnostic test was established and in widespread use. It is assumed that diagnosis levels in 1984 were scaled down from the levels of 1985 as the test was gradually introduced, so that $d_{ki} = c_1 d_{ki}$ where $t_{i¿}$ and t_i correspond to times in 1984 and 1985 respectively. Furthermore, to allow for the diagnoses in the database that pre-date the 1984 introduction of the diagnostic test, a further proportional reduction in the diagnosis probabilities was allowed for to cover this period $d_{ki^{**}} = c_0 c_1 d_{ki}$, for $t_{i^{**}}$ corresponding to calendar times prior to 1984. The parameters c_0 and c_1 are given uninformative flat U[0, 1] priors. The priors for the initial (1985) levels of diagnosis are based on the results of an earlier study.[8]

Estimation

Evaluation of the posterior distributions used for inference was problematic due to their non-standard form, making them algebraically intractable. Their estimation was therefore made possible through Markov Chain-Monte Carlo (MCMC) simulation methods, which were implemented via the JAGS software,[9] embedded into the statistical package R,[10] using the RJAGS packages.[11]

Derived Quantities

Together with the disease progression probabilities, the estimated diagnosis probabilities at a particular time t specify a distribution for what we define to be a "snapshot" of the time-to-diagnosis interval *i.e.* the interval that would be observed were the probabilities of diagnosis held fixed at the time of the snapshot.

Furthermore, in equation (1), we calculate E_{ki} the expected numbers of people in each of the CD4 count states k at the end of the ith time interval. These quantities allow us to estimate the undiagnosed prevalence over time and the time-varying distribution of the undiagnosed prevalence across the CD4 count states. Further detail on how to derive these posterior distributions as well as how the calculate the snapshot time-to-diagnosis distributions can be found in [7].

Results

HIV Incidence

Figure 1 (main paper) shows summaries of the posterior distributions for the number of new infections for each year, 2001-2010. These summaries consist of posterior medians and the corresponding 95% credible intervals. To detect any difference between the levels of incidence in each of the years, and, therefore, to provide evidence of any possible trend, we use posterior probabilities, p_{ij}, that incidence in year i is greater than incidence in year j. These can be readily estimated from the MCMC simulation. Values of p_{ij} that lie close to 0 or 1 provide evidence that there is a significant difference between the incidence in the two years.

For the years under study, the most significant of these posterior probabilities was the one corresponding to the years 2001 and 2004, which stated that incidence was greater in 2004 with probability 0.89. This is not considered to be particularly significant deviation in incidence between the two years. For the last three years, there is much weaker evidence of any difference to any of the other years, as can be expected from the widening credible intervals attached to the incidence over this period. For instance the posterior probabilities $p_{2010,j}$ range from 0.31 (2004) to 0.54 (2001).

Goodness-of-fit

The plots in Figure 2 show the ability of the model to predict the last ten years-worth of quarterly HIV and AIDS at HIV diagnosis counts, as well as the observed distribution of CD4 counts at diagnosis, conditional upon the size of the sample.

The dashed lines represent 95% predictive intervals under the model. As can be seen, these contain almost all (>99%) of all the data points, highlighting the model's suitability for handling such data.

Sensitivity to the Choice of the Incubation Period Distribution

An underlying assumption in the model is that the mean incubation period to AIDS is taken to be 8·6 years, as estimated from a longitudinal analysis of individual CD4 count histories from the CASCADE collaboration.[12] Longer incubation periods have been quoted in literature on the basis of similar types of data,[13,14] with the discrepancy attributable to differences in the assumed distribution of CD4 counts at, or shortly after, seroconversion. If a slower infection progression is assumed, such as the 11 years obtained by a survival analysis,[12] this would simply serve to further smooth the infection incidence curve and not lead to any changes to the trend in HIV incidence, our primary focus. Preliminary work has also suggested our conclusions would not be substantially altered if the model is extended to account for the known differences

in HIV progression associated with age at infection.

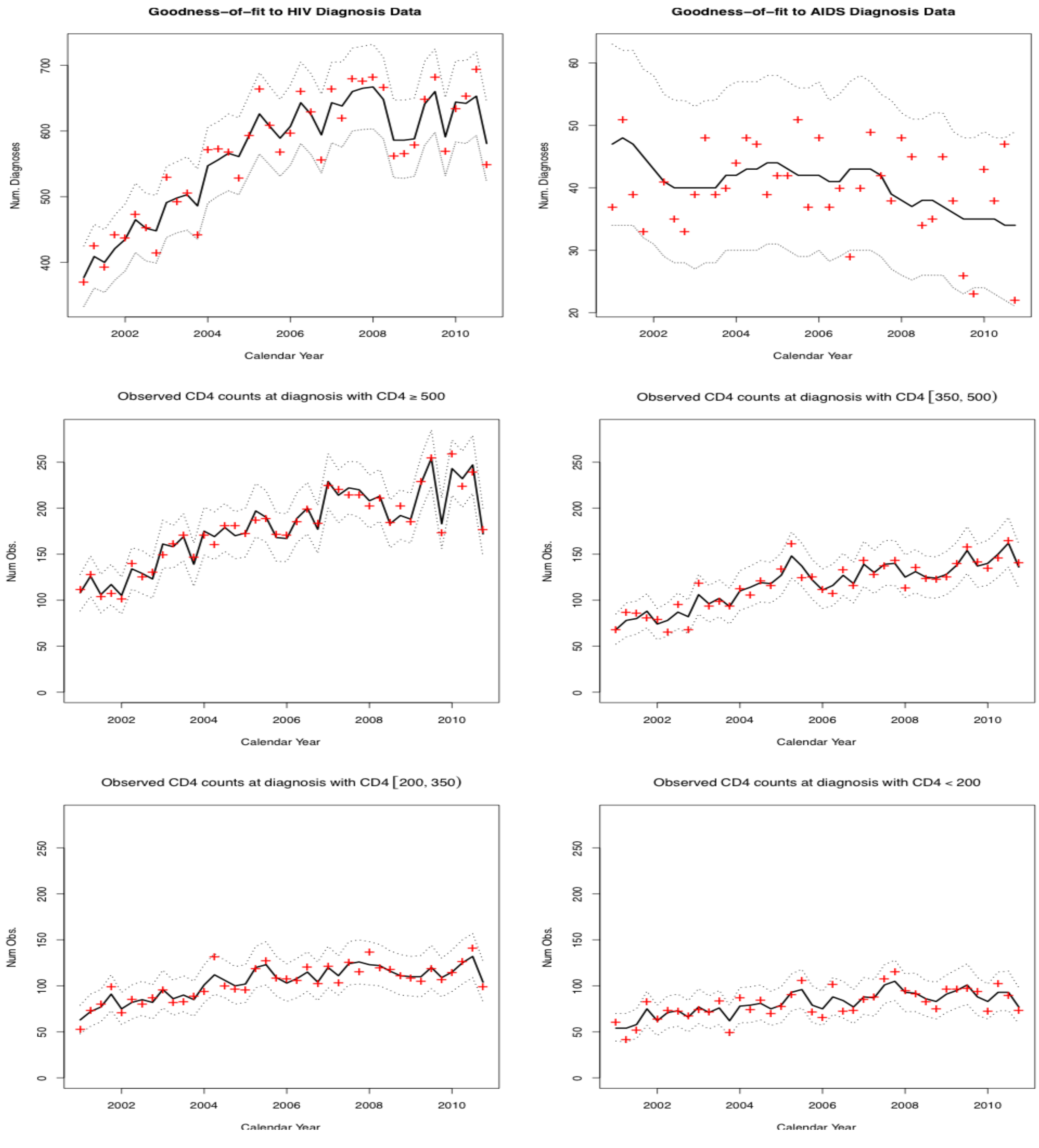

Figure 2*: Goodness-of-fit plots relating the data (red +'s) to the predictive distribution under our model, represented by its median (solid black line) and bounds of the 95% credibility interval (dotted line). Plots are arranged so that the HIV diagnoses are in the top left, the AIDS diagnoses are top right, and the bottom four plots are the CD4 count-at-diagnosis data.*

References

1 Brookmeyer, R & Gail, M. H. AIDS Epidemiology. *Oxford University Press,* 1994.

2 Aalen, O. O, Farewell, V. T, De Angelis, D, & Day, N. E. The Use of Human Immunodeficiency Virus Diagnosis Information in Monitoring the Acquired Immune Deficiency Syndrome Epidemic. *Journal of the Royal Statistical Society (A)* 1994; **157:** 3–16.

3 Marschner, I. C. Using time of first positive hiv test and other auxiliary data in back-projection of aids incidence. *Statistics in Medicine* 1994; **13:** 1959–1974.

4 Yan, P, Zhang, F, & Wand, H. Using HIV Diagnostic Data to Estimate HIV Incidence: Method and Simulation. *Statistical Communications in Infectious Diseases* 2011; **3**.

5 Sweeting, M. J, De Angelis, D, & Aalen, O. O. Bayesian back-calculation using a multi-state model with application to HIV. *Statistics in Medicine* 2005; **24:** 3991–4007.

6 CASCADE Collaboration. Changes in the uptake of antiretroviral therapy and survival in people with known duration of HIV infection in Europe: results from CASCADE. *HIV Medicine* 2000; **1:** 224–231.

7 Birrell, P. J, Chadborn, T. R, Gill, O. N, Delpech, V. C & De Angelis, D. Estimating Trends in Incidence Time-to-Diagnosis and Undiagnosed Prevalence using a CD4-based Bayesian Back-calculation *Submitted*.

8 Aalen, O. O, Farewell, V. T, De Angelis, D, Day, N. E, & Gill, O. N. A Markov model for HIV disease progression including the effect of HIV diagnosis and treatment: application to AIDS prediction in England and Wales. *Statistics in Medicine* 1997; **16:** 2191–2210.

9 Plummer, M. JAGS: Just another Gibbs sampler. 2012 mcmc-jags.sourceforge.net, Accessed 17/10/2012.

10 R Development Core Team. *R: A Language and Environment for Statistical Computing* (R Foundation for Statistical Computing, Vienna, Austria). 2011; ISBN 3-900051-07-0.

11 Plummer, M. *rjags: Bayesian graphical models using MCMC*. R package version 3-2. 2011 http://CRAN.R-project.org/package=rjags, Accessed 17/10/2012.

12 Collaborative Group on AIDS Incubation and HIV Survival including the CASCADE EU Concerted Action. Time from HIV-1 seroconversion to AIDS and death before widespread use of highly-active antiretroviral therapy: a collaborative re-analysis. Lancet 2000 April 1;355(9210):1131–7.

13 Hendriks JC, Satten GA, Longini IM, van Druten HA, Schellekens PT, Coutinho RA et al. Use of immunological markers and continuous-time Markov models to estimate progression of HIV infection in homosexual men. AIDS 1996 June;10(6):649–56.

14 Hendriks JC, Craib KJ, Veugelers PJ, van Druten HA, Coutinho RA, Schechter MT et al. Secular trends in the survival of HIV-infected homosexual men in Amsterdam and Vancouver estimated from a death-included CD4-staged Markov model. Int J Epidemiol 2000 June;29(3):565–72.
